# Assessment of radiation exposure and public health before and after the operation of Sanmen nuclear power plant

**DOI:** 10.3389/fpubh.2023.1131739

**Published:** 2023-02-06

**Authors:** Hong Ren, Shunfei Yu, Ziyou Wang, Taotao Zheng, Hua Zou, Xiaoming Lou, Peng Wang, Lei Zhou, Dongxia Zhang, Meibian Zhang, Jiadi Guo, Zhongjun Lai, Yaoxian Zhao, Zhiqiang Xuan, Yiyao Cao

**Affiliations:** ^1^Zhejiang Provincial Center for Disease Control and Prevention, Hangzhou, Zhejiang, China; ^2^Sanmen County Center for Disease Control and Prevention, Sanmen, Zhejiang, China; ^3^National Institute of Occupational Health and Poison Control, Chinese Center for Disease Control and Prevention, Beijing, China

**Keywords:** Sanmen nuclear power plant, radioactivity, risk assessment, drinking water, ambient dose

## Abstract

**Introduction:**

Sanmen nuclear power plant (SNPP) operates the first advanced passive (AP1000) nuclear power unit in China.

**Methods:**

To assess the radiological impacts of SNPP operation on the surrounding environment and the public health, annual effective dose (*AED*) and excess risk (*ER*) were estimated based on continuous radioactivity monitoring in drinking water and ambient dose before and after its operation during 2014–2021. In addition, the residents' cancer incidence was further analyzed through authorized health data collection.

**Results:**

The results showed that the gross *α* and gross *β* radioactivity in all types of drinking water were ranged from 0.008 to 0.017 Bq/L and 0.032 to 0.112 Bq/L, respectively. The cumulative ambient dose in Sanmen county ranged from 0.254 to 0.460 mSv/y, with an average of 0.354 ± 0.075 mSv/y. There is no statistical difference in drinking water radioactivity and ambient dose before and after the operation of SNPP according to Mann–Whitney *U* test. The Mann-Kendall test also indicates there is neither increasing nor decreasing trend during the period from 2014 to 2021. The age-dependent annual effective doses due to the ingestion of drinking water or exposure to the outdoor ambient environment are lower than the recommended threshold of 0.1 mSv/y. The incidence of cancer (include leukemia and thyroid cancer) in the population around SNPP is slightly higher than that in other areas, while it is still in a stable state characterized by annual percentage changes.

**Discussion:**

The current comprehensive results show that the operation of SNPP has so far no evident radiological impact on the surrounding environment and public health, but continued monitoring is still needed in the future.

## 1. Introduction

To achieve the goal of carbon neutralization by 2060 (1), China has set its national strategy to develop various new energy technologies, and nuclear power development is one of the important means. Currently, there are 55 nuclear power plants (NPPs) in operation and 18 under construction in China (2). With the fast growth of nuclear power industry, the changes of environmental radioactivity levels and associated health impacts receive increasing recognition by the residents in the surrounding areas of NPPs (3–5), especially after the Fukushima Daiichi NPP accident in 2011. To this end, many countries have carried out surveillance programs for radioactivity monitoring and radiological risk assessment before and after the operation of NPPs (6, 7).

Sanmen NPP (SNPP), located in Sanmen county, Zhejiang Province, is the first Chinese nuclear power plant in operation applying advanced passive (AP1000) technology, which is claimed as the safest and most advanced commercial nuclear power technology in the global nuclear power market. However, emission of radioactive debris or effluents into the environment through air and water might be inevitable during its operation. The atmospheric and liquid discharges from an AP1000 reactor estimated by Westinghouse were 1.1 × 10^13^ Bq and 3.3 × 10^13^ Bq, respectively, which are comparable with releases from a 1,000 MWe pressured water reactor in Europe (8). Therefore, it is important to assess the impacts of the operation of SNPP on the radiation safety of the surrounding environment and the local public, and evaluate the merit of AP1000 technology toward radiation protection. To the best of our knowledge, very few data have been reported so far regarding the effects of the operation of AP1000 nuclear power units on the environmental radioactivity and radiation exposure (9). Therefore, it is necessary to track and monitor the radioactivity level of radiation exposure before and after the operation of the nuclear power plant, so as to grasp the radioactivity situation and change trend in time.

Internal and external radiation exposure are the two main ways that radiation affects the health of human body. According to the report of United Nations Scientific Committee on the Effects of Atomic Radiation (UNSCEAR) in 2000 (10), drinking water is considered to be an important factor in increasing radiation exposure to humans. People who are exposed to relatively high levels of radioactivity in drink water and ambient environment for a long period may develop serious health problems, such as cancer (11).

This study was designed to investigate radioactivity levels in drinking water and ambient radiation before and after the operation of the first Chinese AP1000 nuclear power unit from 2014 to 2021. Base on the time-series observation data, the annual effective dose (*AED*) and excess risk (*ER*) of different age groups were estimated. The effect of operation of SNPP on the public health as indicated by the incidence of cancer in the population around the NPP was also evaluated.

## 2. Materials and methods

### 2.1. Sample collection and analysis

#### 2.1.1. Drinking water

**Figure 1:**
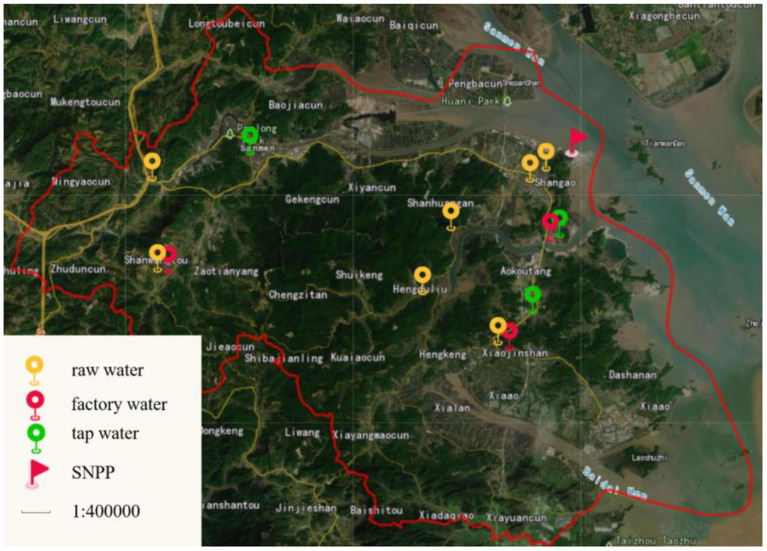
Sampling stations of drinking water around SNPP.

In Sanmen county, raw water is sourced from reservoirs, streams and wells. Tap water is supplied through a complete water supply system for the entire county. In this work, water samples including raw water, factory water and tap water were collected in May and November for each year from 2014 to 2021 at fixed stations around SNPP. The sampling locations for different waters are shown in Figure 1. In total, 92 raw water samples were collected during 2014–2021 from 5 reservoirs, 1 well and 1 stream. In addition, 40 factory water samples from 3 waterworks and 40 tap water samples from the health building of Sanmen country, hospitals and residents' house were collected.

In the sampling collection, 5 L of each water sample was directly transferred to a plastic container, and acidified immediately with 100 ml of nitric acid to minimize the absorption of radioactivity onto the wall of the container. After transporting back to the lab, the sample was evaporated to dryness at 150°C, and calcined at 450°C for 8 h. The residue was then transferred to a sample tray for gross *α* and gross *β* counting. The radioactivity concentrations of gross *α* and gross *β* were measured using the *α*/*β* counting system (BH1217II, China National Nuclear Corporation, China; LB790, Berthold Technologies, Germany), the counting time was 1,000 min.

#### 2.1.2. Ambient radiation

**Figure 2:**
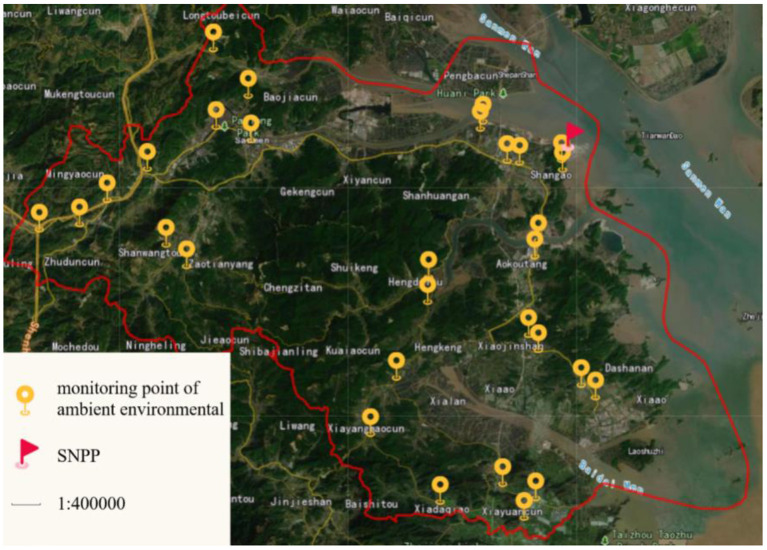
Monitoring stations for ambient radiation exposure of ambient environmental around SNPP.

Being centered around SNPP, in total of 30 stations were set up throughout the Sanmen county except the central mountainous areas for monitoring background radiation level of the ambient environment with thermoluminescence dosimeter (TLD, Beijing Guangrun Yitong Radiation Monitoring Equipment Co. LTD.). Two TLDs were placed at each monitoring site to ensure a high recovery rate. The locations of the monitoring stations are shown in the map in Figure 2. The cumulative ambient dose (CAD) was measured by TLD reader (RGD-3B, Institute of Chemical Defense, China) and the measuring time was 30 s (12).

### 2.2. Quality control and quality assurance

The instruments used in this study, such as low-background *α*/*β* counter and TLDs reader, were inspected by a third-party authoritative metrology agency every two years, and all instruments meet the qualification requirements. ^239^Pu and ^90^Sr–^90^Y plating sources as well as standard source (^241^Am and ^40^K) were used for efficiency calibration before the determination of gross *α* and *β* radioactivity concentrations. And the TLDs were calibrated with a ^137^Cs source before the determination (13).

In order to ensure the analytical quality, 10% of each batch of water sample was selected for repeated analysis to observe the stability and accuracy of the detection method. The lab participated into national inter-comparison exercises organized by the Chinese Center for Disease Control and Prevention (CDC) for gross *α* and gross *β* in water and ambient radiation dose monitoring every year, and satisfied results were achieved.

### 2.3. Statistical analysis

The Mann-Kendall test statistic ***Z*** was adopted using Origin 2021 (Learning Version 9.8) in this study to verify whether there was a monotonic increasing (or decreasing) trend with time in radioactivity of gross *α* and gross *β* in drinking water and background radiation in the ambient environment. A significance level *α* of 0.05 was chosen for the test, if |***Z***| < **Z**_1−*α*/2_, no monotonic trend exists.

The Mann–Whitney *U* test was carried out for the environmental radioactivity levels before and after the operation of SNPP with SPSS 25.0, *P* < 0.05 was considered to indicate a statistically significant difference.

### 2.4. Estimation of annual effective dose and excess risk

Annual effective dose (AED, mSv/y) was adopted to assess the risk to people exposed to internal and external radiation in this study. *AED* due to the ingestion of both gross *α* and gross *β* in drinking water was calculated by Eq. (1).


(1) $$\begin{array}{l} AED_{i}=A\times C\times IR\times T \end{array}$$

Where *A* is the radioactivity concentration of gross *α* and gross *β* (Bq/L); *C* is the age-dependent dose conversion factor for ingestion of radionuclides (mSv/Bq); *IR* is the average daily ingestion rate of drinking water for groups with different ages (L/d); and *T* is the duration of intake, which is 365.25 d.

Since gross *α* radioactivity is mainly due to ^226^Ra and gross *β* radioactivity is due to ^40^K (14, 15), the age-dependent effective dose conversion factors according to ICRP Publication 72, as summarized in Table 1, were used to calculate the effective dose for gross *α* and gross *β* (16, 17). Table 1 also shows the age-dependent values of *IR* in Sanmen county, obtained from the survey conducted by the Ministry of Ecology and Environment of the People's Republic of China (18, 19).

**Table 1:** The age-dependent drinking water ingestion rates, outdoor occupancy factors, and effective dose conversion factors.

**Age (y)**	**1–2**	**2–3**	**3–4**	**4–5**	**5–6**	**6–9**	**9–12**	**12–15**	**15–18**	**≥18**
*IR* (L/d)	0.101	0.899	0.773	0.755	0.822	0.904	0.952	0.981	1.094	1.588
O (d^−1^)	0.115	0.131	0.122	0.100	0.100	0.069	0.059	0.064	0.064	0.133
*C* of ^226^Ra (mSv/Bq)	9.6 × 10^−4^	6.2 × 10^−4^				8.0 × 10^−4^		1.5 × 10^−3^		2.8 × 10^−4^
*C* of ^40^K (mSv/Bq)	4.2 × 10^−5^	2.1 × 10^−5^				1.3 × 10^−5^		7.6 × 10^−6^		6.2 × 10^−6^

*AED* due to background radiation in the ambient environment was calculated using Eq. (2).


(2) $$\begin{array}{l} AED_{e}=CAD\times O \end{array}$$

Where *CAD* is the ambient cumulative dose (mSv); and *O* is the age-dependent outdoor occupancy factor obtained from earlier studies for Chinese population (18, 19), (see Table 1).

The excess risk (*ER)*, which refers to the excess rate of occurrence of a particular health effect associated with radiation exposure, was estimated using Eq. (3).


(3) $$\begin{array}{l} ER=AED\times RF\times DL \end{array}$$

Where *RF* is detriment-adjusted nominal risk factor for cancer and heritable effects after exposure to radiation at a low dose rate, with recommended value of 5.5 × 10^−5^/mSv for cancer and 0.2 × 10^−5^ /mSv for heritable effects by ICRP 103 (20); and *DL* is the duration of life (70 years).

### 2.5. Analysis of cancer incidence

The health data of residents of Sanmen county from 2014 to 2021 were collected from the Zhejiang Provincial Chronic Disease Management System, which is classified according to International Statistical Classification of Diseases and Related Health Problems 10th Revision (ICD-10). The incidences of all cancer sites combined (ICD-10: C00-C97), leukemia caner (ICD-10: C91-95) and thyroid cancer (ICD-10: C73) were analyzed. The leukemia and thyroid cancers were selected for specific investigation because of their radiosensitivity (21).

Crude incidence is calculated by dividing the annual number of cases by the number of people exposed during the same period. In order to enable comparative analysis in the same dimension, we used the Chinese standard population in 2000 and World Segi's population as the base to calculate the age-standardized rates of incidence of China (ASRIC) and the age-standardized rates of incidence of World (ASRIW), respectively. The annual percentage change (APC) was adopted to characterize the temporal trends of incidence, using the Joinpoint model (Version 4.9.0.0).

The collection of health data relied on the Chronic Disease Management System in Zhejiang Provincial Center for Disease Control and Prevention (CDC), which was authorized by Zhejiang provincial government. The analyzing and processing of the data in this study were approved by the Ethics Committee of Zhejiang CDC, in line with the relevant principles of the Declaration of Helsinki, and were carried out in strict accordance with confidentiality requirements during the study.

## 3. Results and discussion

### 3.1. Radioactivity levels in drinking water

The radioactivity concentrations of gross *α* and gross *β* from different sources of drinking water around the SNPP during 2014–2021 are given in Table 2. The activity concentrations of the gross *α* and gross *β* measured in all types of drinking water samples ranged from 0.008 to 0.017 Bq/L and 0.032 to 0.112 Bq/L respectively. The measured gross *α* and gross *β* activities in all water samples were below the WHO recommended thresholds (0.5 Bq/L for gross *α*, 1.0 Bq/L for gross *β*) (22) and generally at the lower end compared to the surveys carried out at a global scale (23–29).

**Table 2:** Radioactivity of gross *α* and gross *β* in three types of drinking water around SNPP from 2014 to 2021.

**Year**	**Raw water**		**Factory water**		**Tap water**	
	**Gross** *α* **(Bq/L)**	**Gross** *β* **(Bq/L)**	**Gross** *α* **(Bq/L)**	**Gross** *β* **(Bq/L)**	**Gross** *α* **(Bq/L)**	**Gross** *β* **(Bq/L)**
2014	0.017 ± 0.011	0.073 ± 0.047	0.013 ± 0.007	0.040 ± 0.002	0.013 ± 0.007	0.035 ± 0.006
2015	0.009 ± 0.003	0.059 ± 0.045	0.008 ± 0.000	0.053 ± 0.028	0.008 ± 0.000	0.034 ± 0.028
2016	0.010 ± 0.006	0.112 ± 0.148	0.008 ± 0.000	0.062 ± 0.033	0.008 ± 0.000	0.082 ± 0.092
2017	0.008 ± 0.000	0.046 ± 0.013	0.008 ± 0.000	0.054 ± 0.008	0.008 ± 0.000	0.041 ± 0.018
2018	0.008 ± 0.000	0.049 ± 0.014	0.008 ± 0.000	0.039 ± 0.013	0.008 ± 0.000	0.032 ± 0.010
2019	0.008 ± 0.000	0.043 ± 0.010	0.008 ± 0.000	0.049 ± 0.023	0.008 ± 0.000	0.034 ± 0.006
2020	0.010 ± 0.005	0.042 ± 0.023	0.008 ± 0.000	0.043 ± 0.010	0.009 ± 0.003	0.040 ± 0.006
2021	0.011 ± 0.008	0.055 ± 0.012	0.008 ± 0.000	0.046 ± 0.006	0.008 ± 0.000	0.049 ± 0.007
Mean ± SD	0.010 ± 0.003	0.060 ± 0.024	0.009 ± 0.002	0.048 ± 0.008	0.009 ± 0.002	0.043 ± 0.016

The average values of gross *α* radioactivity concentrations for three types of water samples in 2014–2021 were 0.010 ± 0.003 Bq/L, 0.009 ± 0.002 Bq/L, and 0.009 ± 0.002 Bq/L, respectively, while the averages of gross *β* were 0.060 ± 0.024 Bq/L, 0.048 ± 0.008 Bq/L, and 0.043 ± 0.016 Bq/L, respectively. Taking uncertainties into account, the average gross *α* or gross *β* radioactivity concentrations were comparable among these three types of drinking water. Compared to the reported gross *α* and *β* radioactivity concentrations in tap waters collected from the surroundings of NPPs in other six provinces (Jiangsu, Shandong, Guangdong, Guangxi, Hainan, and Liaoning) in China, the levels of gross *α* and gross *β* radioactivity in Sanmen tap water were relatively low (see Table 3) (30). The result of this study is consistent with the survey of the Qinshan NPP from 2012 to 2020 (31), confirming the drinking water gross *α* and gross *β* radioactivity in Zhejiang Province is still at the background level.

**Table 3:** Comparison of the radioactivity of gross *α* and gross *β* in tap waters of this study and other studies from different provinces in China (30).

		**Jiangsu**	**Shandong**	**Guangdong**	**Guangxi**	**Hainan**	**Liaoning**	**This study**
Gross *α* (Bq/L)	Range	ND^a^-0.110	ND~0.412	ND−0.110	ND−0.027	ND~0.045	–	0.008~0.011
	Mean ± SD	0.075 ± 0.019	0.169 ± 0.131	0.063 ± 0.033	0.010 ± 0.007	0.012 ± 0.018	0.040	0.009 ± 0.002
Gross *β* (Bq/L)	Range	ND−0.140	ND~0.859	ND−0.420	0.023–0.064	0.012–0.221	–	0.042–0.112
	Mean ± SD	0.101 ± 0.028	0.327 ± 0.276	0.154 ± 0.105	0.045 ± 0.013	0.050 ± 0.036	0.150	0.043 ± 0.016

a ND, Not detectable.

Statistical analysis for the temporal trend over time in the past 8 years indicates that, all the ***Z*** values are <**Z**_0.975_ (see Table 4), suggesting no monotonic increasing (or decreasing) trend exist in the gross *α* and gross *β* activity concentrations of drinking water in the study region from 2014 to 2021.

**Table 4:** Statistical test for the temporal trend of drinking water radioactivity in 2014–2021 around SNPP.

		***Z*** **-value**	***Z*** **_0.975_**	**Trend**
Raw water	Gross *α*	0.169	1.960	No trend
	Gross *β*	−1.523		
Factory water	Gross *α*	−0.508		
	Gross *β*	−0.042		
Tap water	Gross *α*	−0.169		
	Gross *β*	0.508		

The SNPP has been operating continuously for more than 4 years since it started generating power in 2018. Statistical analysis (see Table 5) for comparing drinking water radioactivity levels before (2014–2017) and after (2018–2021) the operation of SNPP shows that there was no significant (*P* > 0.05) change in gross *α* and gross *β* activity concentrations in all three types (raw, factory and tap) of water after the SNPP operation. This suggests that the operation of SNPP had no detectable influence on the radioactivity levels in drinking water around SNPP.

**Table 5:** Statistical test for drinking water radioactivity comparison before and after the operation of SNPP.

**Type of Water**	**Gross** ***α*** **(Mean** ±**SD, Bq/L)**		***Z***	***P***	**Gross** ***β*** **(Mean** ±**SD, Bq/L)**		***Z***	***P***
	**Before** **(2014–2017)**	**After** **(2018–2021)**			**Before** **(2014–2017)**	**After** **(2018–2021)**		
Raw water	0.011 ± 0.007	0.009 ± 0.005	−1.137	0.255	0.048 ± 0.016^a^	0.047 ± 0.016	−0.683	0.495
Factory water	0.009 ± 0.003	0.008 ± 0.000	−1.225	0.221	0.055 ± 0.022	0.044 ± 0.014	−1.740	0.082
Tap water	0.009 ± 0.003	0.008 ± 0.002	−0.329	0.742	0.040 ± 0.014^a^	0.039 ± 0.010	−0.680	0.497

a Some discrete values are eliminated according to Grubbs criterion.

### 3.2. Cumulative ambient dose

Table 6 shows the *CAD* around SNPP during 2014–2021 in Sanmen County, which are ranged from 0.254 to 0.460 mSv, with an average of 0.354 ± 0.075 mSv. The results are consistent with the reported value (1.040 ± 0.044 mSv) in the neighboring Ninghai county, which were carried out prior to the operation of SNPP to monitoring the background radiation levels (32). The average *CAD* around Qinshan NPP reported by Cao (31) and Liu (33), i.e., 0.332 and 0.53 mSv, respectively, are also comparable to the investigation results of this study.

**Table 6:** *CAD* around SNPP and statistical analysis for the temporal trend from 2014 to 2020.

**Year**	***CAD*** **(mSv)**					***Z*** **-value**	***Z***_**0.975**_	**Trend**
	**1st Quarter**	**2nd Quarter**	**3rd Quarter**	**4th Quarter**	**The total**			
2014	0.051	0.055	0.058	0.090	0.254	0.943	1.960	No trend
2015	0.079	0.130	0.063	0.078	0.350			
2016	0.069	0.107	0.073	0.072	0.321			
2017	0.026	0.052	0.083	0.190	0.351			
2018	0.090	0.064	0.090	0.216	0.460			
2019	0.053	0.074	0.080	0.188	0.395			
2020	0.123	0.151	0.099	0.065	0.438			
2021	0.076	0.088	0.059	0.038	0.261			
Mean ± SD	0.071 ± 0.029	0.090 ± 0.036	0.076 ± 0.015	0.117 ± 0.069	0.354 ± 0.075			

Statistical analysis was performed to illustrate the temporal trend based on the quarterly *CAD* data of 30 monitoring stations during 2014–2021 (as shown in Table 6), and the results indicate that ***Z*** = 0.943, **Z**_0.975_ = 1.960, thus |***Z***| < **Z**_0.975_. This indicates that no monotonic trend exists in the *CAD* and the ambient radiation in the study area was kept at a background level during 2014–2021.

The average *CAD* before (2014–2017) and after (2018–2021) the operation of SNPP was 0.319 ± 0.046 mSv and 0.431 ± 0.089 mSv (see Table 7), respectively. In the statistical analysis for comparing the quarterly and annual cumulative doses before and after operation of SNPP, *P*-values were all >0.05, indicating no significant difference between the *CAD* before and after the SNPP operation.

**Table 7:** Statistical analysis for comparison of *CAD* before and after the operation of SNPP.

	***CAD*** **(mSv)**		***Z***	***P***
	**Before** **(2014–2017)**	**After** **(2018–2021)**		
1st quarter	0.056 ± 0.023	0.089 ± 0.029	−1.443	0.149
2nd quarter	0.086 ± 0.039	0.096 ± 0.039	−0.577	0.564
3rd quarter	0.069 ± 0.011	0.090 ± 0.017	−1.155	0.248
4th quarter	0.108 ± 0.056	0.156 ± 0.088	−0.289	0.773
The total	0.319 ± 0.046	0.431 ± 0.089	−1.443	0.149

### 3.3. Age-dependent annual effective dose and excess risk

To assess the potential human health impact of radioactivity in drinking water in the study area, the age-dependent *AED*_*i*_ and *ER*_*i*_ are calculated and summarized in Table 8. The results show that annual effective doses induced by the ingestion of water for the residents in Sanmen county range from 3.30 × 10^−4^ mSv/y to 1.04 × 10^−2^ mSv/y, which are lower than the guideline value of 0.1 mSv/y recommended by WHO ^13^. Therefore, the consumption of drinking water around SNPP would not pose a radiological risk to the local public. The results of this study are generally consistent with previous studies around Qinshan NPP, where *AED* values were ranged from 3.9 × 10^−4^ mSv/y to 9.3 × 10^−3^ mSv/y. The *AED*_*i*_ values obtained in this work are lower than the values in some studies in other countries, such as 0.0209 ~ 2.118 mSv for adults in Western Niger Delta of Nigeria (34), 0.12 ± 0.08 mSv and 0.07 ± 0.05 mSv for adult and child the Capital City of Ekiti State, Nigeria (35) and 0.89 mSv for adult in Jordan (36).

**Table 8:** Age-dependent *AED*_*i*_ and *ER*_*i*_ induced by the ingestion of drinking water (tap water) for the population around SNPP from 2014 to 2021.

**Age (y)**	***AED_***i***_*** **(**×**10**^**−3**^ **mSv/y)**			***ER_***i***_***×**10**^**−5**^		
	**Min**	**Max**	**Avg**	**Min**	**Max**	**Avg**
1–2	0.33	0.72	0.40	0.13	0.29	0.16
2–3	1.85	3.96	2.21	0.74	1.58	0.88
3–4	1.59	3.41	1.90	0.63	1.36	0.76
4–5	1.55	3.33	1.85	0.62	1.33	0.74
5–6	1.69	3.62	2.02	0.67	1.45	0.81
6–9	2.25	4.80	2.66	0.90	1.92	1.06
9–12	2.37	5.06	2.78	0.95	2.02	1.11
12–15	4.39	9.34	5.06	1.75	3.72	2.02
15–18	4.89	10.41	5.65	1.95	4.15	2.25
>18	1.41	3.02	1.67	0.56	1.21	0.67

Based upon the same radioactivity concentrations of drinking water from the same area, the age-distribution pattern of *AED*_*i*_ lies on the different drinking water intake and conversion factors for different age groups. The lowest *AED*_*i*_ was observed in children aged 1–2 years, with an average value of 0.40 × 10^−3^ mSv/y, mainly due to the lowest drinking water intake in this age group. With the increase of age, the drinking water intake gradually increased, consequently increased *AED*_*i*_. The highest *AED*_*i*_ of 5.65 mSv/y was found in the age group of 15–18 years, mainly because the effective dose conversion factor reached the maximum of 1.5 × 10^−3^ mSv/Bq in this group. The corresponding *ER*_*i*_ of *AED*_*i*_ were ranged from 0.16 × 10^−5^ to 2.25 × 10^−5^, and demonstrated the same age-distribution pattern as the *AED*_*i*_. All the obtained *ER*_*i*_ values in this work are much lower than the WHO recommended threshold level (3.99 × 10^−4^) (22).

To assess the potential impact of ambient radiation on the human health, age-dependent *AED*_*e*_ and *ER*_*e*_ are calculated and summarized in Table 9. The obtained averages of *AED*_*e*_ ranged from 21.1 × 10^−3^ mSv/y to 47.5 × 10^−3^ mSv/y. Among the children under 18 years old, the group of children aged 2–3 years spend the longest time outdoor activity, therefore the highest average of *AED*_*e*_ value (46.8 × 10^−3^ mSv/y) was observed in this group. With the age increase, the average daily outdoor activity time for children is constantly reduced, thus the *AED*_*e*_ value is also decreased. The average *AED* per person received from terrestrial radiation (outdoors and indoors) is 0.48 mSv as estimated by UNSCEAR. Thus, the *AED*_*e*_ contributes a marginal proportion to this total terrestrial radiation dose. The average *ER*_*e*_ values were in the range of 9.1 × 10^−5^ ~ 18.9 × 10^−5^, showing the same age-distribution pattern as *AED*_*e*_.

**Table 9:** Age-dependent *AED*_*e*_ and *ER*_*e*_ induced by the exposure of ambient environment for the population around SNPP from 2014 to 2021.

**Age (y)**	***AED_***e***_*****(**×**10**^**−3**^ **mSv/y)**			***ER_***e***_***×**10**^**−5**^		
	**Min**	**Max**	**Avg**	**Min**	**Max**	**Avg**
1–2	29.2	52.9	41.1	11.7	21.1	16.4
2–3	33.3	60.3	46.8	13.3	24.0	18.7
3–4	31.0	56.1	43.6	12.4	22.4	17.4
4–5	25.4	46.0	35.7	10.1	18.4	14.2
5–6	25.4	46.0	35.7	10.1	18.4	14.2
6–9	17.5	31.7	24.6	7.0	12.7	9.8
9–12	15.0	27.1	21.1	6.0	10.8	8.4
12–15	16.3	29.4	22.8	6.5	11.7	9.1
15–18	16.3	29.4	22.8	6.5	11.7	9.1
>18	33.8	61.2	47.5	13.5	24.4	18.9

### 3.4. Cancer incidence in the vicinity of SNPP

#### 3.4.1. Incidence of all cancers combined

As shown in Table 10, for all cancers combined, a total of 815 new cases were recorded for the residents around SNPP from 2014 to 2021, with a crude incidence of 428.22/100,000, including 438.73/100,000 in males and 416.68/100,000 in females, respectively. This consistent with the incidence rate of inhabitants living around Qinshan NPP (31). The ASIRC of both sexes range from 378.80/100,000 to 498.04/100,000, and the ASIRW of both sexes range from 247.08/100,000 to 302.80/100,000. These values seem to be slightly higher than the incidence of all cancers combined in Zhejiang Province (ASIRC: 229.76/100,000; ASIRW: 220.96/10,000) (37) which may be related to the dietary habits of the local residents. In general, the incidence of all cancers combined for the residents around SNPP was stable over the study period (2014–2021), with a slight upwards trend observed for females (ASIRC: APC = 2.8%; ASIRW: APC = 2.5%).

**Table 10:** Incidence of all cancers combined in residents around SNPP from 2014 to 2021(1/100,000).

**Year**	**Males**				**Females**				**Both sexes**			
	**New cases**	**Crude rate**	**ASIRC**	**ASIRW**	**New cases**	**Crude rate**	**ASIRC**	**ASIRW**	**New cases**	**Crude rate**	**ASIRC**	**ASIRW**
2014	987	428.60	306.78	301.66	807	385.79	273.75	256.12	1,794	408.22	288.28	277.02
2015	914	395.81	276.76	272.20	734	349.59	240.31	226.14	1,648	373.80	256.12	247.08
2016	1,011	436.66	296.80	292.04	842	399.40	274.68	259.50	1,853	418.90	282.72	272.98
2017	978	420.15	276.89	273.54	783	368.80	248.03	235.20	1,761	395.65	260.50	252.52
2018	1,019	436.03	282.51	278.79	899	421.38	283.32	264.37	1,918	429.04	279.71	268.74
2019	1,175	502.43	314.42	311.91	1,053	493.24	319.13	299.45	2,228	498.04	313.46	302.80
2020	1,045	447.30	273.43	267.59	998	468.11	320.73	295.72	2,043	457.23	292.69	277.73
2021	1,030	442.10	259.44	255.77	946	445.18	286.34	263.66	1,976	443.57	268.93	256.45
Total	8,159	438.73	284.99	280.83	7,062	416.68	280.00	262.04	15,221	428.22	279.51	268.77
APC (%)	–	1.5	−1.2	−1.2	–	3.9	2.8	2.5	–	2.6	0.7	0.5
95% *CI* (%)	–	−0.8, 3.9	−3.5, 1.1	−3.6, 1.2	–	0.9, 7.1	−0.4, 6.1	−0.6, 5.7	–	0.1, 5.3	−1.9, 3.3	−2.1, 3.2

#### 3.4.2. Incidence of radiosensitive cancer

Earlier studies showed increased rates of leukemia and thyroid cancer in nearby residents from normal operations or accidents, such as people in the vicinity of German NPPs and Three Mile Island NPP in the United States (38, 39). In this study, we found that a total of 392 new leukemia cases were reported from 2014 to 2021 for the residents around SNPP, with a crude incidence of 11.03/100,000, an ASIRC of 8.59/100,000, and an ASIRW of 8.91/100,000, (see Table 11). The ASIRC of leukemia was stable over the period with the 0.2% of APC (95% *CI*: −6.8% to 7.7%), and kept at the same level as that in Zhejiang Province from 2010 to 2014 (ASIRC: 5.26/100,000; ASIRW: 5.60/100,000) (40).

**Table 11:** Leukemia and thyroid cancer incidences in residents around SNPP from 2014 to 2021 (1/100,000).

**Year**	**Leukemia**				**Thyroid cancer**			
	**New cases**	**Crude rate**	**ASIRC**	**ASIRW**	**New cases**	**Crude rate**	**ASIRC**	**ASIRW**
2014	57	12.97	10.33	10.63	183	41.64	31.91	28.17
2015	41	9.30	7.04	7.08	170	38.56	30.33	26.76
2016	45	10.17	8.25	9.22	190	42.95	34.33	30.86
2017	34	7.64	6.85	7.29	185	41.56	33.27	29.80
2018	48	10.74	8.56	8.96	200	44.74	36.78	32.27
2019	63	14.08	11.09	11.18	249	55.66	44.49	39.49
2020	57	12.76	9.35	9.37	244	54.61	45.45	39.36
2021	47	10.55	7.36	7.59	221	49.61	41.73	35.33
Total	392	11.03	8.59	8.91	1,642	46.20	37.14	32.68
APC (%)	–	1.7	0.2	−0.2	–	4.6	5.9	5.3
95%*CI* (%)	–	−6.0, 10.2	−6.8, 7.7	−6.9, 7.0	–	1.4, 8.0	2.9, 8.9	2.2, 8.5

A total of 1,642 new thyroid cancer cases were reported for the residents around SNPP in 2014–2021, with a crude incidence rate of 46.20/100,000, an ASIRC of 37.14/100,000, and an ASIRW of 32.68/100,000. These values are slightly higher than the incidence in China (ASIRC: 12.05/100,000; ASIRW:10.44/10,000) (41), and show a slight upward trend (ASIRC: APC = 5.9%,; ASIRW: APC = 5.3%), which is consistent with that observed in China and other countries (42–46). There are many factors contributing to the increase in thyroid cancer incidence, such as ionizing radiation, iodine intake, female hormones, and body mass index (BMI) (47). It is also likely related to the availability and improvement of thyroid gland imaging examination techniques (47, 48), and may even reflect “overdiagnosis” through increased use of new imaging technologies (49, 50), thus increasing the detection of thyroid cancer cases. However, the increase rate of the incidence of thyroid cancer in residents of Sanmen county from 2014 to 2021 is less than the relevant data reported in Zhejiang Province (48) (APC = 28.62%) and nationwide in China (APC = 12.4%) (51), and the incidence is controllable at present.

The health effects of radiation on residents around the NPP need to be observed over a long period of time. Due to the short operation time of SNPP, the current investigation results have not indicated any impact on the health of residents around the NPP. The radiation safety for the environment and human health in the surrounding area of SNPP was secured in the past years, with the successful application of advanced AP1000 technology in SNPP. We acknowledge that the present study may not enable us to identify and quantify the merits of AP1000 technology in terms of radiation protection, e.g., possibly less radioactive releases during the operation compared to traditional nuclear power technologies. Continued monitoring for the public health state and further investigations on the spatiotemporal distribution of some specific radionuclides (such as fission products) in the surrounding environment of SNPP would be needed to fill our knowledge gap.

## 4. Conclusions

This study investigated the radioactivity levels of drinking water, background radiation in the ambient environment, and cancer incidence status in the residents around SNPP from 2014 to 2021. The results showed that radioactivity concentrations of gross *α* and gross *β* in all types of drinking water were lower than the recommended values by WHO, and the measured ambient environmental accumulated doses were all at background levels. Statistical analysis indicated neither monotonic changing trend over 2014–2021, nor statistical difference does before and after the operation of SNPP in the drinking water gross *α* and gross *β* radioactivity and ambient environmental accumulated. Cancer incidences for the residents in Sanmen county was slightly higher than that in other areas, while it was kept relatively stable for all cancers combined and leukemia cancer over the period of 2014–2021. The current comprehensive results show that the operation of SNPP has so far no evident radiological impact on the surrounding environment and public health, but continued monitoring is still needed in the future.

## Data availability statement

The original contributions presented in the study are included in the article/supplementary material, further inquiries can be directed to the corresponding author.

## Ethics statement

The studies involving human participants were reviewed and approved by the Ethics Committee of Zhejiang Provincial Center for Disease Control and Prevention. Written informed consent to participate in this study was provided by the participants' legal guardian/next of kin.

## Author contributions

Manuscript writing and final approval of the manuscript: HR and YC. Manuscript writing: ZW and SY. Final approval of the manuscript: HZ and XL. Collection and detection: PW, LZ, DZ, JG, and ZL. Organize and coordinate: MZ, YZ, and ZX. All authors have read and agreed to the published version of the manuscript.
